# Understanding Loneliness in an Aging Population of San Vito de Coto Brus, Costa Rica

**DOI:** 10.5334/aogh.4586

**Published:** 2024-12-30

**Authors:** Nicholas Leahy, Melissa Rallo, Lillianna Pedersen, Christine Wan, Hima Konduru, Shania Bailey, Alexis Vetack, Wendel Mora, Shailvi Gupta, Carlos Faerron Guzmán

**Affiliations:** 1 University of Maryland School of Medicine, Baltimore, MD, USA; 2 Centro Interamericano para la Salud Global (CISG), Puntarenas, Costa Rica; 3 Hands for Health, Puntarenas, Costa Rica; 4 Center for Global Engagement, University of Maryland Baltimore, Baltimore, MD, USA

**Keywords:** aging, loneliness, social determinants of health

## Abstract

*Introduction:* As trends in life expectancy continue to improve, the burden of loneliness in geriatric populations on a global scale is increasing. With advancing age comes an increase in the number of life events that can perpetuate a state of loneliness such as losing a life partner, dwindling social networks, and deteriorating health conditions. This burden can manifest in a variety of mental and physical consequences. While loneliness has been studied in a few communities around the world, there is a need to study loneliness in the context of Latin American communities in Central America, including Costa Rica. The aim of the present study is to assess the prevalence and associated factors of social and emotional loneliness (SEL) in a sample of elderly patients in the canton of Coto Brus, Costa Rica.

*Methods:* A cross‑sectional study was conducted that sampled 63 adults aged 65 years or above in the canton of Coto Brus. Investigators conducted face‑to‑face interviews in Spanish with the aid of translators. The primary instruments used for the present study were a content‑validated version of the 11‑item De Jong Gierveld Loneliness Scale and socio‑demographic questions that included age, sex, address, civil status, and level of education.

*Results:* A high degree of SEL was found, with 60.3% of participants noting at least a moderate degree of loneliness, with the average score being 3.33 on the 11‑point scale. When SEL was broken up into its respective subscores, the average score for social loneliness (SL) was found to be 0.67 on the 5‑point scale, and the average score for emotional loneliness (EL) was found to be 2.67 on the 6‑point scale. There was also evidence that supports both level of education and marital status serving as protective factors in the development of SEL.

*Discussion:* These results could indicate a stronger association of loneliness being linked to missing a life partner compared with loneliness being linked to having smaller social networks. Given the associations that were found in this preliminary study, it is pivotal to explore loneliness in this community with a larger sample size—potentially through the integration of the country’s Equipos Básicos de Atención Integral de Salud (EBAIS) healthcare system. It is also crucial to expand the study to explore any associations between loneliness and comorbid mental and physical health conditions.

## 1. Introduction

Loneliness among older adults has garnered increased attention as shifting demographic trends and societal dynamics shape experiences of aging on a global scale. While research has been established to assess the relationship between loneliness and the aging process in the United States and various European countries [1–3], there is a lack of research that has sought to understand this relationship among older adults in Latin America.

Loneliness has been traditionally understood as a subjective state that contrasts with the condition of physical isolation, implying an imbalance in the desired and achieved level of socio‑affective interaction [4]. In an aging population, the loss of a spouse and diminishing social networks due to death of friends or reduced community integration are common triggers of loneliness [5]. However, it is also critical to consider other factors that play a role in the subjective experience. Research from a Latin American study highlights urbanization of rural areas, retirement from careers, declining birth rates, departure of sons and daughters from the home, and the pervasive influence of technology that potentially isolates older adults in their homes as factors that can precipitate a state of loneliness. The study goes on to say that, while these may be associated with independence in some cultures, in a Latin American environment these may be considered risk factors that potentially alienate older members from a community [6].

Frameworks from prior literature such as Disengagement Theory, which posits that adults intrinsically and responsively reduce the amount of social interactions they have as they age, help to give relevance to behavioral patterns that are seen with old age. The withdrawal from societal roles and interactions has widespread effects on levels of social connectivity that lead to exacerbated feelings of isolation [7]. Personality traits such as poor social skills, shyness, and introversion can further predispose individuals to social isolation, complicating efforts to maintain robust social networks crucial for well‑being in later life. Conversely, higher levels of education and economic resources often serve as protective factors against loneliness, affording individuals greater opportunities for social engagement and support [8].

The distinction between social isolation, defined as minimal contact with others, and loneliness, characterized by the subjective experience of lacking a meaningful social network, is crucial [9]. Loneliness, whether emotional (lack of a close attachment) or social (absence of a broader social network), is increasingly recognized for its association with adverse health outcomes, including high risks of chronic diseases, depression, and decreased quality of life among older adults in Latin America [4]. These implications underscore the urgent need for comprehensive strategies that not only connect older adults to community resources but also address their holistic well‑being through nutrition, physical activity, and spiritual support [10].

**Figure 1:**
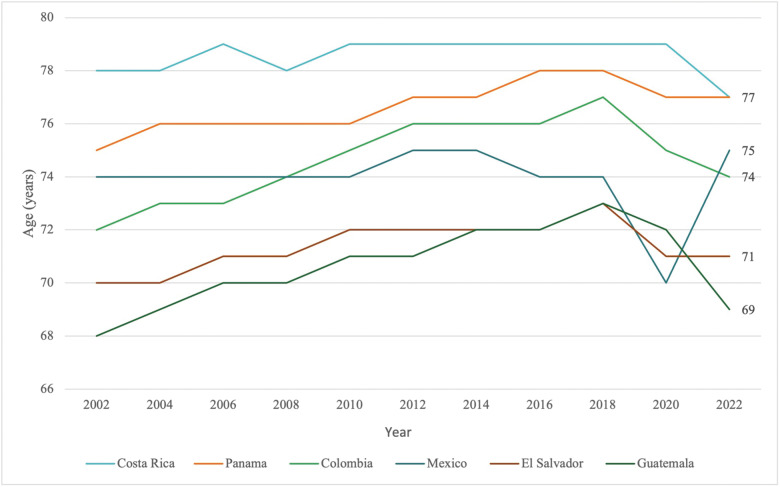
Life expectancy at birth—countries in Latin America. Source for Demographic Data: World Bank Group 2024.

The principles of loneliness from prior literature, outlined above, were used in the present study that sought to understand social and emotional loneliness (SEL) in San Vito de Coto Brus, Costa Rica. Trends in life expectancy for Costa Rica exceed other upper‑middle‑income countries (UMICs), including those in Latin America (Figure 1) [11]. A study that contrasted health risk factors between Costa Rica and the United States, a country with a life expectancy of 77 years [11], found lower rates of smoking, obesity, hypertension, and single‑person households in Costa Rica could contribute to this characteristic despite having a much lower GDP per capita compared with the United States [12]. Yet, even with trends of higher life expectancy, it is important to recognize that healthy aging is multifactorial, and understanding how loneliness is implicated in this process is critical, as the burden of elderly populations in countries such as Costa Rica continues to increase. The prevalence of care dependency, or the degree of difficulty that people have performing regular activities of daily living, is about 10% for individuals over 65 years in Costa Rica, with the level of dependency only increasing with age. [13] And with about 12% of the population being over the age of 65 years in Costa Rica [11], this only perpetuates issues such as familial abandonment and involuntary placement in long‑term care facilities [6].

Additionally, the presence of cultural norms and gender roles, particularly a patriarchal ideology prevalent in Costa Rican society, shape how loneliness is perceived and experienced by older adults. Men, conditioned to be competitive and less emotionally expressive, may struggle more with loneliness due to societal expectations that hinder their ability to seek companionship when needed [14]. While women tend to outlive men and experience higher rates of widowhood, this could manifest as greater feelings of loneliness and lower quality of life, as evidenced by a study of loneliness in China, India, and Latin American countries such as Cuba, Venezuela, and Mexico [15]. Yet, there is counterevidence to show that women, traditionally educated in empathy and caretaking roles, may find it easier to seek and provide social support, potentially mitigating feelings of loneliness through communal ties and spiritual practices—especially in the strong Catholic communities that are characteristic of rural Costa Rica, including San Vito de Coto Brus [16].

Understanding the intricate interplay of demographic, cultural, and psychological factors contributing to loneliness among older Latin American populations is essential for developing effective interventions and policies. Assessing the relationship in the context of a rural community of Costa Rica can help contribute to the framework of loneliness among aging populations in Latin American countries.

## 2. Ethical Considerations

The study was approved by the institutional review board (IRB) at the University of Maryland, Baltimore (HP‑00105818) and by Costa Rica’s Comité Ético Científico Fundación Instituto Costarricense de Investigación y Enseñanza en Nutrición y Salud (CEC‑FUNIN; approved in session FIDE‑CEC‑099‑2023). Informed consent was given and signed prior to conducting interviews. Interviews were only conducted after participants agreed to participate once they were informed of the details and goals of the present study. Data from these interviews were not shared outside of the research team for scientific purposes.

## 3. Methods

### 3.1 Participants

A cross‑sectional study was conducted that sampled 63 adults aged 65 years or above in the canton of Coto Brus in Costa Rica. Convenience sampling was used to recruit participants for the study, as local contacts in the town of San Vito were used to connect with community groups and nursing homes for a length of data collection that lasted 4 weeks. Investigators conducted face‑to‑face interviews in Spanish with the aid of local translators. Two healthcare professionals from San Vito and San José served as translators throughout the duration of data collection. Translators were present at the time of the interviews and translated the script from English to Spanish in real time.

### 3.2 Measures

The primary instruments used for the present study were a content‑validated version of the 11‑item De Jong Gierveld Loneliness Scale [17], and sociodemographic questions that include age, sex assigned at birth, address, civil status, and level of education. The De Jong Gierveld Loneliness Scale was used for this study because the investigators were interested in differentiating feelings of missing an intimate relationship (emotional loneliness) from the feelings of missing a wider social network (social loneliness). Prior literature has demonstrated that the primary instrument has been validated in two different studies: one that looked at the reliability and validity of the 6‑item abbreviated instrument in France, Germany, the Netherlands, Russia, Bulgaria, Georgia, and Japan [3], and another that looked at the reliability and validity of the 11‑item instrument in Peru [18] and other Spanish‑speaking countries [19, 20]. It was critical to find evidence of the instrument employed in a variety of populations—especially among Latin American populations—to provide evidence of reliability and validity in populations with different languages, cultures, and values. Information of all study participants was collected anonymously, and each participant was given a de‑identification code to ensure data remained anonymous.

### 3.3 Data analysis

The standard scoring system available for the De‑Jong‑Gierveld Loneliness Scale was used to compute scores for participants in the study. For items 2, 3, 5, 6, 9, and 10, responses of “More or less” or “Yes’’ would receive a score of 1 for that item. Items 1, 4, 7, 8, and 11 needed to be reverse‑scored, so a response of “No” would receive a goal of 1 for that item. After summing the total from the collection of 11 items for each participant, overall social‑emotional loneliness (SEL) was categorized into not lonely (0‑2), moderately lonely (3‑8), severely lonely (9‑10), or extremely lonely (11) [17].

## 4. Results

A total of 63 individuals aged 65 years or older were sampled in the canton of Coto Brus, Costa Rica. In addition to the De Jong Gierveld scale for loneliness, general demographic characteristics were collected for the sample (Table 1). In an analysis of the breakdown of the averaged composite score of total loneliness (TL) (3.37), emotional loneliness (EL) comprised a much larger proportion (2.69; 79.82%) compared with social loneliness (SL; 0.68; 20.18%), indicating that the loss of one close attachment during the aging process can have a profound effect on the individual evaluation of loneliness (Table 2). Most of the study participants (*n* = 35) classified themselves as moderately lonely, with a score on the De Jong Gierveld Scale falling between 3 and 8. Among the remaining participants in the study, most of them scored in the category of not lonely (*n* = 25), whereas there were a few participants who scored in the category of severely lonely (*n* = 3). There were no participants who scored in the category of extremely lonely (score = 11 on the De Jong Gierveld Scale).

**Table 1:** Demographic factors of study participants.

CHARACTERISTICS	NUMBER (*N* = 63)	PERCENTAGE
**Age (years)**		
65–74	41	65.08%
75 and above	22	34.92%
**Sex assigned at Birth**		
Male	21	33.33%
Female	42	66.67%
**Marital status**		
Married	30	47.62%
Single/Separated/Widowed	33	52.38%
Highest level of education*		
Incomplete primary school	24	38.71%
Complete primary dchool	38	61.29%

*N = 62 for level of education.

**Table 2:** TL, EL, and SL of study participants.

**Mean participant scores**		**Value**	
TL		3.37	
SL		0.68	
EL		2.69	
**Scores by subcategory**		**Number**	
Not lonely (0–2)		25 (39.68%)	
Moderately lonely (3–8)		35 (55.56%)	
Severely lonely (9–10)		3 (4.76%)	
Extremely lonely (11)		0	
**Loneliness with demographic**	**TL**	**SL**	**EL**
**Factors**			
**Age (years)**			
65–74	3.146	0.634	2.512
75 and above	3.682	0.727	2.955
**Sex assigned at birth**			
Male	3.071	0.476	2.595
Female	3.857	1.048	2.810
**Marital status**			
Married	2.967	0.500	2.467
Not married	3.667	0.818	2.848
**Highest Level of Education**			
Incomplete primary school	4.417	1.000	3.417
Complete primary school	2.579	0.395	2.184

**Figure 2:**
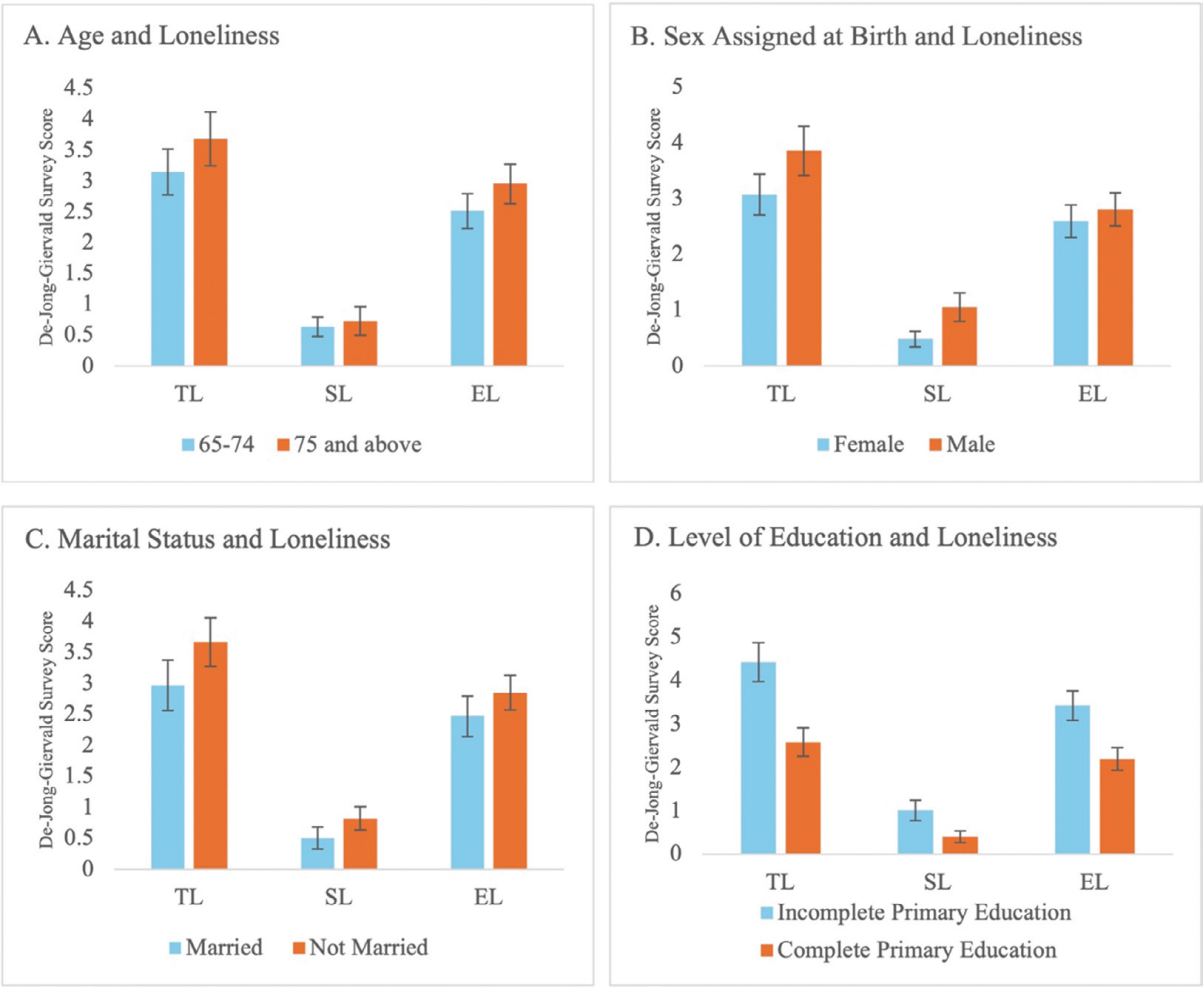
Loneliness with demographic factors. (**A‑D**) TL, SL, and EL are displayed with relation to four different demographic characteristics: (**A**) age with 41 participants falling into the 65‑74 age range while 22 participants fell into the 75 and above age range; (**B**) sex assigned at birth with 21 participants assigned male at birth and 42 participants assigned female at birth; (**C**) marital status with 30 participants who are married and 33 participants who are not married (widowed, separated, single, etc.); and (**D**) level of education with 24 participants who did not complete primary education and 38 participants who have completed primary education. *n* = 63 for each graph except for (**D**) where *n* = 62. Bar graphs are displayed with SEM.

As a part of the preliminary analysis, the relationships between TL, EL, and SL were explored with each individual demographic factor. Given the age distribution of participants in the study, two distinct age categories (65–74 years and 75 years and above) were used for analysis as opposed to regression. While there appears to be a slight positive association of increasing age with the loneliness score on the De Jong Gierveld survey (Figure 2A), especially among TL and EL, a more consistent distribution of individuals over the age of 65 years would be needed to draw any statistical conclusions. When sex assigned at birth was compared with loneliness, there also appears to be a positive association between male sex and loneliness score (Figure 2B). While this association is supported in the literature, researchers ran into the issue of only sampling 21 males compared with 42 females. Once again, a more equal distribution of males to females in the sample would provide more information from which to draw any statistical conclusions. The next demographic factor, marital status, was divided into two categories: married and not married. The ‘not married’ category was composed of single (*soltero*), divorced (*divorciado*), widowed (*viudo*), separated (*separado*), and free union (*unión libre*) individuals. Participants who were married in the sample were only found to have slightly lower loneliness scores for TL, SL, and EL (Figure 2C). And lastly, level of education provided the strongest association with lower loneliness levels. Prior literature has substantiated education as a protective factor in the development of loneliness, and the data in this preliminary analysis support this claim with evidence that level of education has stronger associations with lower loneliness levels compared with age, sex assigned at birth, and marital status (Figure 2D).

## 5. Discussion

The study investigates loneliness among an elderly population in the canton of Coto Brus, Costa Rica, focusing on the distinction between loneliness associated with missing a life partner (EL) versus loneliness linked to having a smaller social network (SL). The findings suggest a potentially stronger association with EL, implying a potential need for targeted support strategies. However, the study acknowledges several methodological limitations, primarily stemming from convenience sampling and the possible bias toward individuals with larger social networks.

One of the notable strengths of the study lies in the use of a validated instrument with demonstrated reliability and validity across multiple countries [3, 18‑20]. The study’s methodology benefits from the simplicity and clarity of the survey instrument, making it easy to administer and score. This approach enhances the feasibility of conducting similar studies in other communities or expanding the sample size to draw more robust statistical conclusions.

The reliance on convenience sampling poses significant limitations. By sampling from community groups and nursing homes, the study may inadvertently exclude elderly individuals who experience severe loneliness and therefore do not actively participate in community activities. This could skew results toward a population with stronger social support networks, potentially underestimating the prevalence and impact of loneliness among the broader elderly population in Coto Brus. Additionally, the community‑centric nature of Coto Brus, where as in many other rural regions in Costa Rica, family plays a pivotal role [16], raises questions about whether the survey adequately captures the nuances between support from family versus friends. This distinction could influence how loneliness is perceived and experienced by individuals in this cultural context, suggesting a need for tailored survey questions that reflect these dynamics more accurately.

The findings underscore important implications for local community leaders in Coto Brus. While existing community outlets may provide everyday fellowship and support, additional targeted interventions are necessary to assist elderly community members coping with the loss of a close emotional attachment, such as a life partner or a significant friend. This could involve bolstering support services that specifically address bereavement and emotional resilience among the elderly. Community leaders could also work to bolster support for education, which the data showed to be one of the strongest protective factors in the development of loneliness. While this may not address the factions of older adults experiencing loneliness potentially due to the effects of a lower level of education over the course of a lifetime, emphasizing the protective nature of education against the development of loneliness, including the other comorbid mental health and chronic health conditions associated with loneliness, will yield positive returns for generations in the future.

This study points toward several avenues for future research. Firstly, expanding the sample size beyond convenience sampling methods would enhance the generalizability of findings. This would allow for more robust statistical analyses, exploring potential associations between loneliness and demographic factors such as age, sex assigned at birth, and marital status. Furthermore, future studies should delve into the relationship between loneliness and its potential impact on comorbid mental and physical health conditions among the elderly population in Coto Brus. Understanding these interrelationships could inform comprehensive healthcare strategies that address both the emotional and physical well‑being of older adults.

One way to analyze the relationship of loneliness with comorbid mental and physical health conditions associated with older age would be to use the current EBAIS model of healthcare in Costa Rica. The establishment of the EBAIS model on a national scale in 1995 helped to revolutionize the delivery of healthcare throughout the country. The tenets of the EBAIS model, which include primary care, accountability, monitoring, and community involvement, have revolutionized what healthcare access can look like. Monitoring through the EBAIS model has also yielded an increase in the burden of noncommunicable diseases since 1990 as the proportion of individuals above 65 years old continues to increase [21]. Given the association of loneliness in older adults with a variety of noncommunicable diseases, assessment of loneliness in rural regions of the country through local EBAIS clinics could be critical as we continue to build our understanding of the relationship between loneliness and aging in Costa Rica.

Endeavors in the future that work to connect loneliness to its dynamic web of causes and comorbidities can help local community leaders better tailor support services to meet the diverse needs of their elderly population, while helping the community as a whole deepen our understanding of loneliness in aging populations not just in Costa Rica but in other Latin American communities as well.

## Data Availability

The authors confirm that the data supporting the findings of this study are available within the article. If further details are needed, please email Nicholas Leahy, the corresponding author (nleahy@som.umaryland.edu).

## Additional File

The additional file for this article can be found as follows:

Supplementary Appendix. 11‑item De Jong Giervald Survey in Spanish.
