# Supplementary material for: Understanding Loneliness in an Aging Population of San Vito de Coto Brus, Costa Rica
Source: Ann Glob Health. 2024 Dec 30;90(1):83. doi: 10.5334/aogh.4586 (PMC11697617; doi:10.5334/aogh.4586)
Supplement: Supplementary Appendix. — 11‑item De Jong Giervald Survey in Spanish. [file agh-90-1-4586-s1.pdf]

Supplementary Appendix

11-item De-Jong-Giervald Survey in Spanish

Statement	No (0)	More or Less (1)	Yes (2)
Siempre hay alguien con quien puedo hablar de sus problemas diarios (+)<br>(There is always someone I can talk to about my day-to-day problems)			
Echo de menos tener un buen amigo / a de verdad<br>(I miss having a really close friend)			
Siento una sensación de vacio a mi alrededor<br>(I experience a general sense of emptiness)			
Hay suficientes personas a las que puedo recurrir en caso de necesidad (+)<br>(There are plenty of people I can rely on when I have problems)			
Echo de menos la compañia de otras personas<br>(I miss the pleasure of company of others)			
Pienso que mi círculo de amistades es demasiado limitado<br>(I find my circle of friends and acquaintances too limited)			
Tengo mucha gente en la que confiar completamente (+)<br>(There are many people I can trust completely)			
Hay suficientes personas con las que tengo una amistad muy estrecha (+)<br>(There are enough people I feel close to)			
Echo de menos tener gente a mi alrededor<br>(I miss having people around)			
Me siento abandonado/a a menudo<br>(I often feel rejected)			
Puedo contar con mis amigos / se siempre que lo necesito (+)<br>(I can call on my friends whenever I need them)			

Scale: No (0), More or Less (1), Yes (2)
